# BRCA2 Status Alters the Effect of the P53 Reactivator HO-3867 in Ovarian Cancer Cells

**DOI:** 10.26502/jcsct.5079197

**Published:** 2023-04-27

**Authors:** Eric J Devor, Ariane E Thomas, Brandon M Schickling

**Affiliations:** 1 Department of Obstetrics and Gynecology, University of Iowa College of Medicine, Iowa, USA; 2 Holden Comprehensive Cancer Center, University of Iowa Hospitals and Clinics, Iowa, USA; 3 Department of Anthropology, University of Iowa, Iowa, USA

**Keywords:** PEO1, PEO4, Ovarian cancer, TP53, BRCA2, Mutation, P53 reactivation

## Abstract

The vast majority of ovarian cancers have a TP53 mutation. Among these, a substantial proportion also have a BRCA1 and/or a BRCA2 mutation. Given a rising interest in the therapeutic use of p53 reactivating agents, we assessed the effect that such BRCA mutants would have on the action of a p53 reactivator.

As an initial tool to examine the effect of a BRCA mutation on the action of a p53 reactivator we chose to utilize a naturally occurring experimental model. The high grade serous ovarian cancer cell lines PEO1 and PEO4 were established from the same patient. Both cell lines have a missense TP53 mutation, G244D. However, PEO1 cells also have a nonsense BRCA2 mutation, Y1655ter, which is cancelled out by a second mutation, Y1655Y, that renders PEO4 cells BRCA2 wild-type. This makes these cell lines an ideal experimental platform to begin to assess the effect of a BRCA mutation on the action of a p53 reactivator.

Both PEO1 and PEO4 cells were treated with a p53 reactivator, the synthetic curcumin analog HO-3867. The effect of treatment was assessed through quantitative PCR (qPCR) assays of fourteen known p53 target loci, including p53 itself. In all cases there was a definite difference between treated and untreated cells relative to their BRCA2 status.

While these results are preliminary, the fact that BRCA2 status influences the effect of a p53 reactivator on numerous target loci suggests that this relationship should be further investigated and that, in future, the BRCA status of ovarian tumors containing missense TP53 mutations should be considered when opting for the therapeutic use of a p53 reactivator.

## 1. Introduction

The tumor suppressor TP53 is widely regarded as the most mutated locus in human cancers [1]. Nowhere is this more true than in ovarian cancer where it is estimated that well over 90% of ovarian tumors harbor a TP53 mutation [2]. Of these, roughly two-thirds are missense mutants located primarily in the DNA binding-domain. Such mutations are attractive targets for the therapeutic application of so-called “p53 reactivators” which include PRIMA-1 and PRIMA-1met (APR246), arsenic trioxide (ATO) and the synthetic curcumin analog HO-3867 [3–6].

It is also well established that up to one-quarter of all high grade serous ovarian cancers (HGSOC) are home to a BRCA1 and/or BRCA2 mutation [7]. Moreover, in a study that examined 98 HGSOC tumors with a BRCA1 and/or BRCA2 mutation, nearly all of them (95) also had a TP53 mutation [8]. This could suggest that up to 25% of HGSOC tumors with a TP53 mutation will also contain a BRCA1 and/or a BRCA2 mutation. In order to determine if this is indeed the case, we searched the AACR Project GENIE database (aacr.org) and found that a significant proportion of ovarian tumors with a BRCA1 and/or a BRCA2 mutation also had a TP53 mutant compared with tumors that were BRCA1 and BRCA2 wild-type (85.9% versus 69.1% respectively, p<0.001). In both BRCA mutant and wild-type tumors, more than half had targetable TP53 missense mutants. Thus, in this context we wondered what effect BRCA status might exert on a p53 reactivator.

To begin to answer this question we chose to use a naturally occurring BRCA2/TP53 experimental model. Cell lines PEO1 and PEO4 were established from ascites effused nine months apart from a patient presenting with a poorly differentiated Stage III ovarian adenocarcinoma [9]. PEO1 cells have a G244D TP53 missense mutation and a Y1655ter BRCA2 mutant rendering these cells BRCA2 NULL. PEO4 cells retain the G244D TP53 missense mutation but a second mutation at Y1655 of BRCA2, Y1655Y, restores wild-type BRCA2 function [10]. This establishes the two cell lines as an ideal pair through which to examine the effect of BRCA2 status on reactivation of a TP53 missense mutant.

We have treated PEO1 and PEO4 cells with the p53 reactivator HO-3867 and herein report that BRCA2 status does in fact alter the effect of such exposure on a variety of loci including TP53 itself. Our results, while preliminary, suggest that BRCA status is a factor to consider in treating HGSOC patients with a p53 reactivator.

## 2. Materials and methods

### 2.1 GENIE database

A total of 3585 ovarian cancer entries were downloaded from the AACR Project GENIE database (aacr.org) in August of 2020. These cases were filtered for the presence of a BRCA1 and/or a BRCA2 mutation and/or a TP53 mutation which yielded a total of 2987 entries. These entries were then filtered for TP53 and BRCA mutations.

### 2.2 Cell culture

PEO1 (CVCL_2686) and PEO4 (CVCL_2690) cells were purchased from the European Collection of Authenticated Cell Cultures (ECACC), items numbered 10032308 and 10032309. Cells were grown in RPMI-1640 media supplemented with 10% fetal bovine serum (R&D systems FBS S11150) and 1% pen/strep antimicrobial (Thermo Fisher 15140122) at 37°C and 5% CO2. Cell line identities were confirmed by verifying the relevant BRCA2 and TP53 mutations via direct sequencing of gDNAs on an Applied Biosystems Model 3730xl capillary sequencer in the Genome Core of the Iowa Institute of Human Genetics (IIHG) and by CODIS panel typing at bioSynthesis (Lewisville, Texas).

### 2.3 HO-3867 p53 reactivator treatment

The synthetic curcumin analog HO-3867 was purchased from Cayman Chemical (Ann Arbor, Michigan, Item Number 21581). PEO1 and PEO4 cells were grown to 80% confluence in T75 vented flasks (Falcon 353136). Six-well culture dishes (Falcon 353046) were then seeded in RPMI-1640 media in triplicates at a density of 200,000 cells per well and incubated at 37°C, 5% CO_2_ for twenty-four hours. Following incubation, media was removed and replaced with fresh RPMI-1640 media containing either vehicle only or 5uM HO-3867. This dosage was chosen on the basis of our previous studies of this agent [11], which was subsequently supported by an independent study [12]. Cells were then incubated for 24 hours at 37°C, 5% CO_2_.

Treated and untreated control cells were collected, and total cellular RNA was purified using the QIAGEN RNeasy Plus Mini Kit (QIAGEN 74134) according to manufacturer’s recommendations. RNA yield and purity was determined using a NanoDrop spectrophotometer (Thermo Fisher).

A fixed mass of 100 ng of total RNA per assay was reverse transcribed in the presence of SuperScript III reverse transcriptase (Invitrogen First Strand SSIII Kit 18080–400). Quantitative PCR (qPCR) assays were then carried out in triplicate on an Applied Biosystems QuantStudio 5 platform in the presence of Applied Biosystems Power SYBR Green Master Mix (Thermo Fisher 437659) using the primers shown in Table 1. All primers were synthesized at Integrated DNA Technologies (Coralville, Iowa).

Raw Ct values for each qPCR assay were normalized (ΔCt) against 18S rRNA (Table 1) and fold changes between HO-3867 treated cells and controls for each assay were calculated using the standard ΔΔCt method [13,14]. Fold change significance was assessed via a two-tailed t-test with unequal variance [15]. A p-value of 0.05 was considered as significant.

## 3. Results

### 3.1 Wild-type p53 is significantly less common in BRCA mutated ovarian cancers

**Figure 1:**
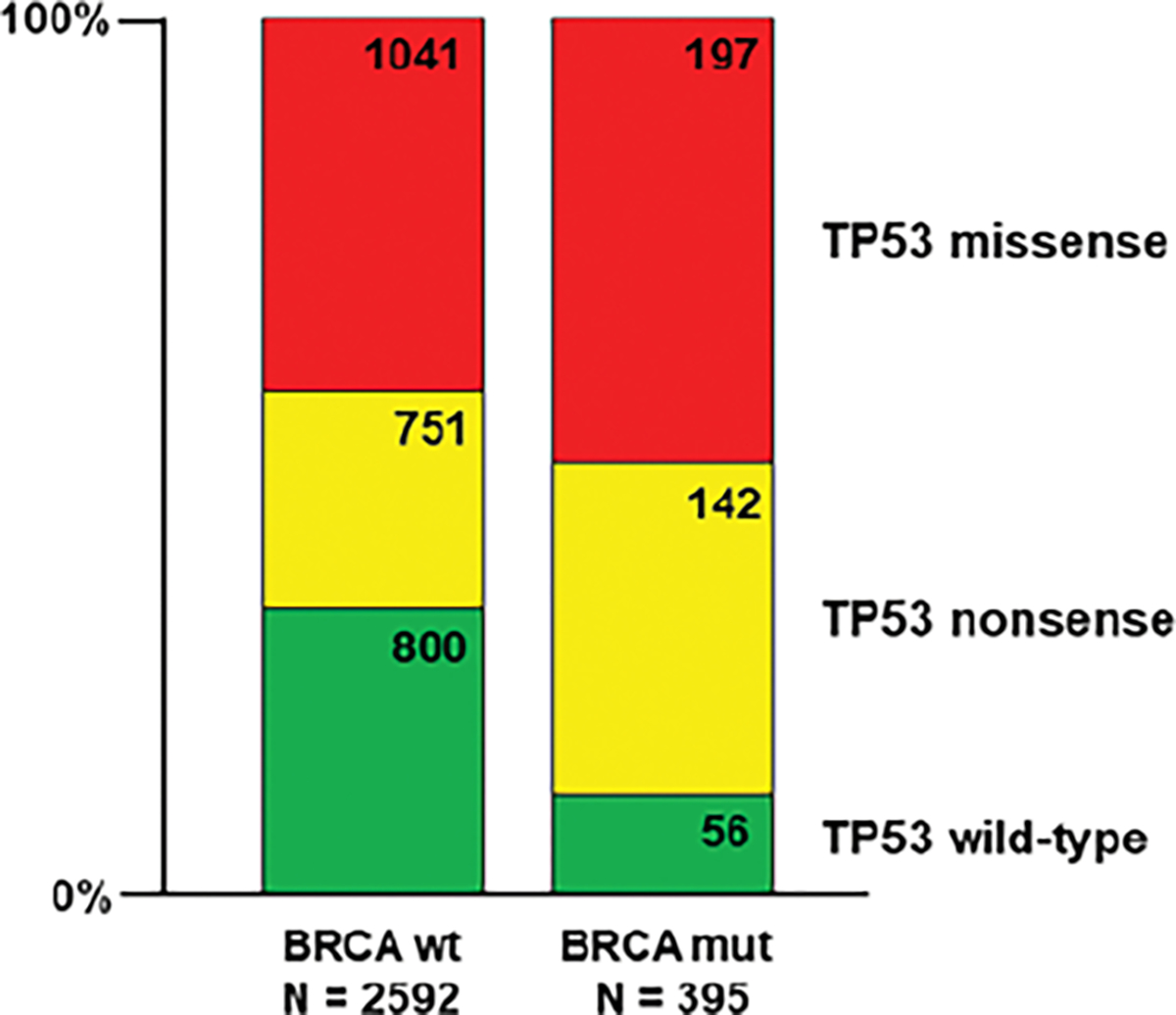
Distribution of TP53 mutations sorted by BRCA1/2 mutation status among 2987 ovarian cancer entries in the AACR Project GENIE data base. A statistically significant difference, driven by the proportion of TP53 wild-type entries, is seen (chi-square = 46.7, p<0.0001, df = 2)

As noted, Boyarskikh et al. [8] reported that 95 of 98 HGSOC tumors containing a BRCA1 or BRCA2 mutation also had a TP53 mutation. More broadly, Dr. Elizabeth Swisher of the University of Washington noted that wild-type TP53 was virtually absent in ovarian cancers with BRCA mutations but relatively common, ~30%, in BRCA wild-type tumors (Personal Communication). We are able to confirm this tendency in our survey of nearly three thousand ovarian tumors culled from the AACR Project GENIE database (Figure 1). As can be seen, 56 of the 395 tumors with a BRCA1 and/or BRCA2 mutation were also TP53 wild-type (14%). By contrast, among the 2592 tumors that were BRCA wild-type, 800 were also TP53 wild-type (31%). However, among the two classes of TP53 mutation the proportion of missense versus nonsense mutants is an identical 58% to 42%. Therefore, the overall difference between the two BRCA classes is exclusively due to the relative dearth of wild-type TP53 in the BRCA mutant class (chi-square = 46.7, p<0.0001, df = 2). Given these data, it should be expected that ovarian tumors, especially HGSOC, harboring a BRCA mutation will also have a TP53 mutation. Conversely, as high as 20% - 25% of ovarian tumors, the vast majority of which will have a TP53 mutation, will also have a BRCA mutation. Of these, nearly 60% will be a targetable missense TP53 mutant.

### 3.2 BRCA status alters the effect of p53 reactivation by HO-3867

In order to assess the effect of BRCA2 status on p53 reactivation we carried out fourteen SYBR Green qPCR assays on PEO1 and PEO4 cells treated with HO-3867, a p53 reactivator that is also an antioxidant [16]. The loci selected for these assays consisted of several consensus p53 target genes including survivin (BIRC5), cyclin D1 (CCND1), p21 (CDKN1A), heme oxygenase 1 (HO-1), NAD(P)H quinone dehydrogenase 1 (NQO1), pleckstrin homology like domain family A member 3 (PHLDA3), placenta-specific protein 1 (PLAC1), TNF receptor superfamily member 10b (TNFRSF10B), TP53 regulated inhibitor of apoptosis 1 (TRIAP1) and zinc finger matrin-type 3 (ZMAT3) [17–23]. In addition, the co-regulated apoptosis pathway gene p38-delta (MAPK13) and two important redox genes NADPH oxidase 4 (NOX4) and NFE2 like BZIP transcription factor 2 (NRF2) were included. Finally, because TP53 is self-regulated via a well-known feedback loop it, too, was included [24].

The results of the 28 qPCR assays are presented in Table 2. First, only four of the 28 comparisons between HO-3867 treated and untreated cells failed to yield a statistically significant change in expression. Three of these, BIRC5, NRF2 and PHLDA3, were in the BRCA2 NULL PEO1 cells while the fourth was in the PEO4 cells for NQO1. With the sole exception of ZMAT3, which showed increased transcription in PEO1 cells and decreased transcription in PEO4 cells, the differences between PEO4 and PEO1 cells were a matter of degree rather than kind. That is, both cell lines displayed an increase in transcription (CDKN1A, HO-1, NQO1, NRF2, TNFRSF10B) or both cell lines displayed a decrease in transcription (BIRC5, CCND1, MAPK13, NOX4, PHLDA3, PLAC1, TP53, TRIAP1). In each case the responses were consistent with the expectation that loci known to be up-regulated by p53 displayed increased transcription and those known to be down-regulated by p53 displayed decreased transcription.

**Figure 2:**
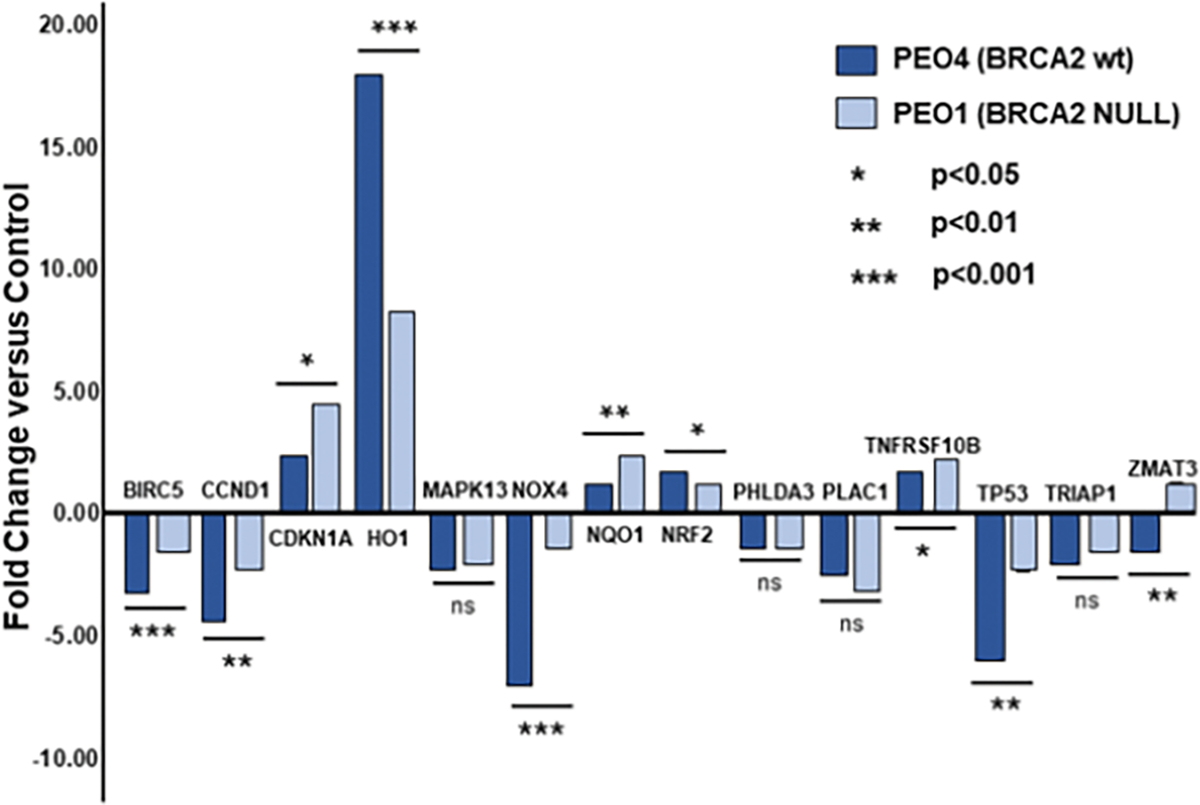
Fold change differences between HO-3867 treated and untreated PEO1 and PEO4 ovarian cancer cells for fourteen qPCR assays. Statistical significance was calculated between cell lines based upon within cell line ΔΔCt values.

The relative effect of HO-3867 exposure in the PEO1 and PEO4 cells is shown graphically in Figure 2. As seen, the effect of HO-3867 treatment on transcription, whether positive or negative, was greater in the BRCA2 wild-type PEO4 cells in nine of the fourteen loci (BIRC5, CCND1, HO-1, MAPK13, NOX4, NRF2, PHLDA3, TP53, and TRIAP1) while it was less in only four of the fourteen loci (CDKN1A, NQO1, PLAC1 and TNFRSF10B). While some of the discrepancies are small, several of the loci show quite large and statistically significant effects. In particular, NOX4 shows five times the effect of HO-3867 exposure in the BRCA2 wild-type PEO4 cells compared with the BRCA2 NULL PEO1 cells (p<0.001). Similarly, HO-1 displays more than double the already high transcriptional response in PEO4 cells compared with PEO1 cells (p<0.001). Finally, PEO4 cells showed nearly two and one-half times the reduction in transcription of TP53 itself compared with PEO1 cells (p<0.01).

## 4. Conclusion

Consistent with a previous study [6], results presented here indicate that treatment with the p53 reactivator HO-3867 rescues p53 target gene transcription. We also show, using BRCA2 NULL PEO1 ovarian cancer cells and the revertant BRCA2 wild-type PEO1 cell line from the same patient, that such rescue is influenced by BRCA2 status. Clearly, our results are from a unique pair of cell lines and the true effect of BRCA1 and/or BRCA2 mutation status on the efficacy of p53 reactivators must be further studied. On the other hand, data from the Cancer Genome Atlas (TCGA) and other sources have shown that as high as 95% of ovarian cancers harbor a TP53 mutation [1,2]. Of these, roughly 60% are missense mutants that are targetable by a p53 reactivator. Further, depending upon histology, as much as one-fourth of these will also have a BRCA1 and/or a BRCA2 mutation. World-wide, more than 300,000 cases of ovarian cancer are diagnosed yearly [25]. The data above suggest that more than 180,000 of these cases will have a targetable TP53 missense mutation but also that nearly 50,000 of these cases will also have a BRCA1 and/or a BRCA2 mutation. This implies that, to be maximally effective, the use of a p53 reactivator should take both TP53 and BRCA1/2 status into account.

## Figures and Tables

**Table 1:** Primer sequences for the assays carried out in this study. All primers were designed against cDNA sequences in GenBank using PrimerQuest software from Integrated DNA Technologies. Each assay was validated by qPCR prior to use in this study.

Assay	Primer Sequences	Amplicon	Tm(°C)
BIRC5	For: CCGCAGTTTCCTCAAATTCTTTC	227bp	54.7
(survivin)	Rev: CTTGGCCCAGTGTTTCTTCT		55.3
			
CCND1	For: GTTCGTGGCCTCTAAGATGAAG	223bp	55.3
(cyclin D1)	Rev: GTGTTTGCGGATGATCTGTTTG		54.9
			
CDKN1A	For: TGAGTTGGGAGGAGGCA	226bp	55.8
(p21)	Rev: GCTTCCTCTTGGAGAAGATCAG		54.9
			
HO1	For: CCAGGCAGAGAATGCTGAGTTC	144bp	57.9
	Rev: AAGACTGGGCTCTCCTTGTTGC		59.6
			
MAPK13	For: CTCACCCATCCCTTCTTTG	118bp	52.9
(p38δ)	Rev: TGTGCTGCTTCCATTCATC		53.1
			
NOX4	For: AACACCTCTGCCTGTTCATC	87bp	55
	Rev: GATACTCTGGCCCTTGGTTATAC		54.6
			
PHLDA3	For: AGCTGTGGAAGCGGAAG	160bp	55.2
	Rev: GTCACCAGCGTGAAGTAGAT		54.4
			
PLAC1	For: CACCAGTGAGCACAAAGCCACATT	232bp	60.3
	Rev: CCATGAACCAGTCTATGGAG		52.3
			
TNFRSF10B	For: GACCCTTGTGCTCGTTGT	175bp	55.4
	Rev: GCAATCTCTACCGTCTTCTGAG		54.8
			
TP53	For: GACCGGCGCACAGAGGAAGAGAAT	110bp	62.9
	Rev: TGGGGAGAGGAGCTGGTGTTGTTG		62.9
			
TRIAP1	For: GAGTACGACCAGTGCTTCAAT	157bp	54.7
	Rev: CCATGAACTCCAGTCCTTCAATA		54.3
			
ZMAT3	For: CCAGGCTCATTATCAGGGTAAA	219bp	54.3
	Rev: GAACTGAAGGAGGCATCACA		54.7
			
18S rRNA	For: AACCTTTCGATGGTAGTCGCCG	104bp	59.4
	Rev: CCTTGGATGTGGTAGCCGTTT		57.6

**Table 2:** HO-3867 treated PEO1 and PEO4 cells compared to untreated controls for fourteen SYBR Green qPCR assays. Fold change was determined via the standard ΔΔCt method while p-value was assessed using a two-tailed t-test with unequal variances. p<0.05 is significant.

		Fold Change	
Cell Line	Assay	Versus Untreated	p-value
PEO4	BIRC5	−3.23	0.001
PEO1	BIRC5	−1.39	0.062
			
PEO4	CCND1	−4.35	0.001
PEO1	CCND1	−2.17	0.001
			
PEO4	CDKN1A	2.3	0.001
PEO1	CDKN1A	4.52	0.001
			
PEO4	HO-1	17.79	0.001
PEO1	HO-1	8.23	0.001
			
PEO4	MAPK13	−2.27	0.001
PEO1	MAPK13	−1.75	0.001
			
PEO4	NOX4	−6.67	0.001
PEO1	NOX4	−1.35	0.048
			
PEO4	NQO1	1.18	0.222
PEO1	NQO1	2.21	0.001
			
PEO4	NRF2	1.69	0.004
PEO1	NRF2	1.09	0.416
			
PEO4	PHLDA3	−1.28	0.049
PEO1	PHLDA3	−1.1	0.686
			
PEO4	PLAC1	−2.38	0.001
PEO1	PLAC1	−3.03	0.001
			
PEO4	TNFRSF10b	1.67	0.002
PEO1	TNFRSF10b	2.22	0.001
			
PEO4	TP53	−5.88	0.001
PEO1	TP53	−2.33	0.001
			
PEO4	TRIAP1	−1.96	0.002
PEO1	TRIAP1	−1.47	0.001
			
PEO4	ZMAT3	−1.52	0.015
PEO1	ZMAT3	−1.3	0.021

## Data Availability

Data used in this study are available from the authors upon reasonable request.
